# ICTV Virus Taxonomy Profile: *Nairoviridae*

**DOI:** 10.1099/jgv.0.001485

**Published:** 2020-08-25

**Authors:** Aura R. Garrison, Sergey V. Alkhovsky [Альховский Сергей Владимирович], Tatjana Avšič-Županc, Dennis A. Bente, Éric Bergeron, Felicity Burt, Nicholas Di Paola, Koray Ergünay, Roger Hewson, Jens H. Kuhn, Ali Mirazimi, Anna Papa [Άννα Παπά], Amadou Alpha Sall, Jessica R. Spengler, Gustavo Palacios, ICTV Report Consortium

**Affiliations:** ^1^​ USAMRIID, Fort Detrick, Frederick, Maryland, USA; ^2^​ D. I. Ivanovsky Institute of Virology of N. F. Gamaleya National Center on Epidemiology and Microbiology of Ministry of Health of Russian Federation, Russia; ^3^​ University of Ljubljana, Ljubljana Faculty of Medicine, Slovenia; ^4^​ UTMB, Galveston, Texas, USA; ^5^​ Viral Special Pathogens Branch, Division of High-Consequence Pathogens and Pathology, CDC, Atlanta, Georgia, USA; ^6^​ Division of Virology, National Health Laboratory Service and Division of Virology, University of the Free State, Bloemfontein, Republic of South Africa; ^7^​ Virology Unit. Department of Medical Microbiology, Faulty of Medicine, Hacettepe University, Ankara, Turkey; ^8^​ Public Health England, Porton Down, UK, Wiltshire, Salisbury; ^9^​ Integrated Research Facility at Fort Detrick. NIAID, NIH, Fort Detrick, Frederick, Maryland, USA; ^10^​ Folkhalsomyndigheten, Stockholm, Sweden; ^11^​ National Reference Centre for Arboviruses and Haemorrhagic Fever viruses, Department of Microbiology, Medical School, Aristotle University of Thessaloniki, Thessaloniki, Greece; ^12^​ Institut Pasteur de Dakar, Dakar, Senegal

**Keywords:** *Bunyavirales*, bunyavirus, *Nairoviridae*, nairovirus, ICTV Report, orthonairovirus, shaspivirus, striwavirus, taxonomy

## Abstract

Members of the family *Nairoviridae* produce enveloped virions with three single-stranded RNA segments comprising 17.1 to 22.8 kb in total. These viruses are maintained in arthropods and transmitted by ticks to mammals or birds. Crimean-Congo hemorrhagic fever virus is tick-borne and is endemic in most of Asia, Africa, Southern and Eastern Europe whereas Nairobi sheep disease virus, which is also tick-borne, causes lethal haemorrhagic gastroenteritis in small ruminants in Africa and India. This is a summary of the International Committee on Taxonomy of Viruses (ICTV) Report on the family *Nairoviridae*, which is available at ictv.global/report/nairoviridae.

## Virion

**Fig. 1.**
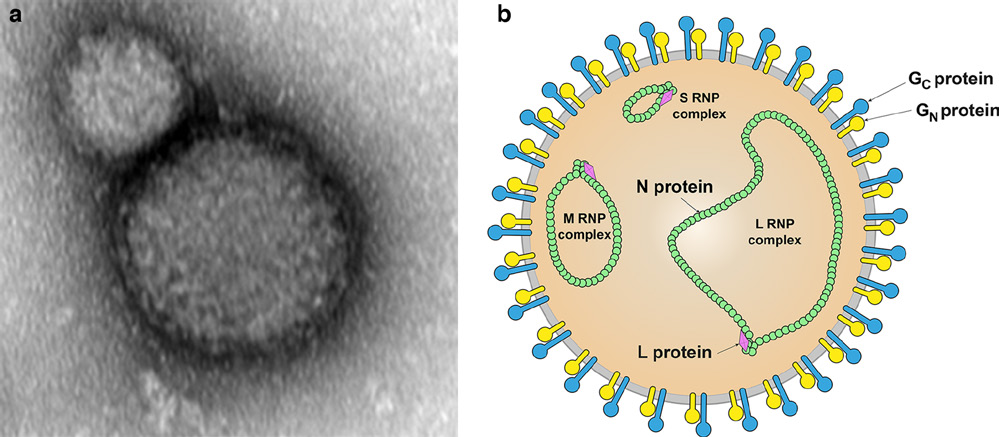
(a) Transmission electron micrograph of a Crimean-Congo hemorrhagic fever virus particle. (b) Schematic illustration of a nairovirus particle.

Where known, virions are spherical in shape, 80–120 nm in diameter with a membrane envelope decorated with glycoprotein (GP) spikes composed of G_N_ and G_C_ (Table 1, Fig. 1). Isolated ribonucleoprotein (RNP) complexes are composed of individual segments of genomic RNA encapsidated in nucleocapsid (N) protein. The RNPs are associated with large (L) protein.

**Table 1.** Characteristics of members of the family *Nairoviridae*

Typical member:	Dugbe virus [S segment: AF434161; M segment: M94133; L segment: U15018], species *Dugbe orthonairovirus*, genus *Orthonairovirus.*
Virion	Enveloped, spherical virions 80–120 nm in diameter with heterodimer surface spikes
Genome	Three single-stranded, negative-sense RNA molecules, S, M, and L of about 2 kb, about 5 kb, and about 12 kb, respectively
Replication	Cytoplasmic. The nucleocapsid protein (N) encapsidates the genomic RNA forming ribonucleoprotein (RNP) complexes with the viral RNA-directed RNA polymerase (RdRP)-containing large protein (L). Anti-genomic RNAs are generated and serve as templates for synthesis of nascent RNP complexes containing genomic RNA
Translation	From capped mRNAs that lack poly(A) termini. The 5′-cap structure is derived from cellular mRNAs via cap-snatching
Host range	Birds, humans, rodents, hares, shrews, ruminants, bats, ticks (*Orthonairovirus*); spider vector (*Shaspivirus*) or water strider vector (*Striwavirus*) with unknown host range
Taxonomy	Realm *Riboviria*, phylum *Negarnaviricota*, class *Ellioviricetes*, order *Bunyavirales*; several genera and >15 species

## Genome

**Fig. 2.**
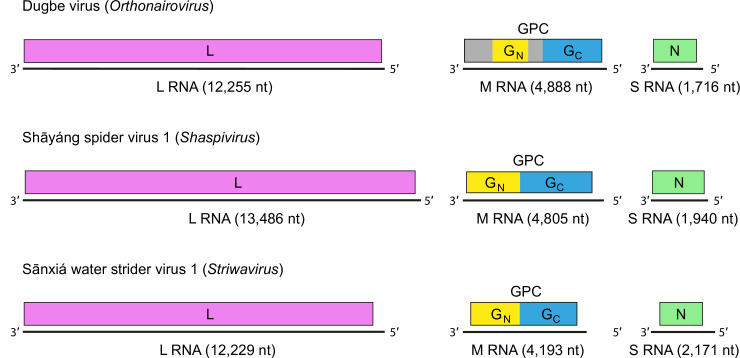
Schematic representation of nairovirus genome organization.

The nairovirus genome (Fig. 2) consists of two to three single-stranded, negative-sense RNA molecules, termed S (small), M (medium; if present), and L (large). These RNAs encode respectively, in the virus-complementary sense, N, the GP precursor (GPC), and L (containing RdRP, helicase, and endonuclease domains).

## Replication

Virions attach to unknown cell-surface receptors and enter via the endosomal route [1]. Viral fusion with the host cell results in early or late endosomal release of the virion RNP complex into the cytoplasm. This pH-dependent fusion event likely requires the previous participation of an intracellular receptor [1, 2]. During primary transcription the virion-associated L protein generates antigenomic RNAs, which are capped using host-cell-derived capped primers [3]. Translation is by free (L and S segment mRNAs) or membrane-bound (M segment mRNA) ribosomes. Based on evidence from Crimean-Congo hemorrhagic fever virus, GPC is co-translationally cleaved to yield glycosylated G_N_ and G_C_ and non-structural glycoproteins [4]. Antigenome RNA synthesized by the RdRP domain of the L protein serves as a template for genomic RNA replication. Secondary transcription amplifies the synthesis of mRNAs and genome replication. During morphogenesis, G_N_ and G_C_ accumulate in the Golgi, are terminally glycosylated, modified host membranes are acquired, and the virions bud into the Golgi cisternae [5, 6].

## Taxonomy

Current taxonomy: ictv.global/report/nairoviridae. Nairoviruses form a family in the polyploviricotine order *Bunyavirales*, and are most closely related to members of the family *Wupedeviridae*. Like most other bunyaviruses, nairoviruses (i) have multisegmented, negative-sense single-stranded RNA genomes; (ii) encode proteins with high sequence identity; (iii) have five conserved motifs (A–E) in their RdRP domain; and (iv) produce enveloped virions.

## Resources

Current ICTV Report on the family *Nairoviridae*: ictv.global/report/nairoviridae
